# Neurological manifestations in coronavirus disease 2019 (COVID-19) patients: a systematic review of literature

**DOI:** 10.1017/S1092852920001935

**Published:** 2020-10-21

**Authors:** Wael Ibrahim

**Affiliations:** Department of Neurology, Kasr Alainy Hospital, Faculty of Medicine, Cairo University, Cairo, Egypt

**Keywords:** COVID-19, corona virus, SARS-CoV2, ARDS, neurological manifestations, systematic review

## Abstract

**Background:**

The exact incidence of neurological complications from coronavirus disease 2019 (COVID-19) infection remains unknown. Neurological symptoms are more common with severe form of the disease. Through neuro-invasion, the virus can affect both neurons and glial cells and induce wide range of neurological pathologies.

**Objectives:**

To systematically assess the neurological manifestations in patients diagnosed with COVID-19.

**Methods:**

A systematic literature search of the PubMed, Scopus, and Cochrane databases was performed. Randomized controlled trials, nonrandomized controlled trials, observational studies of neurological manifestations in patients diagnosed with COVID-19.

**Results:**

All three-database search identified 89 publications. A total of 22 full-text articles assessed for eligibility with 12 articles excluded. Altogether, the included studies reported 290 patients with neurological manifestations. Neurological manifestations were subdivided into central causes (CNS) and peripheral causes (PNS). CNS symptoms is commoner representing 91% of all neurological patients with 9% only with PNS. Headache represented the commonest neurological symptoms in regard to number of patients, meanwhile dizziness has the highest incidence with 11.9%. Neurological manifestations were divided according to COVID-19 severity into: (1) nonsevere and (2) severe; with all CNS manifestations were more in severe patients except headache were more in nonsevere patients. All included studies were on adult patients except one study in pediatric patients with limited number of participants.

**Conclusions:**

From the descriptive analyses and available data of relatively small sample-sized studies, it can be concluded that in spite of the aforementioned limitations, that a wide spectrum of neurological manifestations including CNS and PNS can occur in COVID-19 patients.

## Introduction

Coronavirus (CoV) represents a large family of RNA viruses found in different animal species including birds, livestock, and mammals such as bats. These virsues are known to affect different human systems including the respiratory, hepatic, nervous, and gastrointestinal systems. Subtypes of CoV which are known to be pathogenic to humans usually cause mild clinical symptoms except for two subtypes; severe acute respiratory syndrome related coronavirus (SARS-CoV) and Middle East respiratory syndrome coronavirus (MERS-CoV).^1^

**Figure 1.**
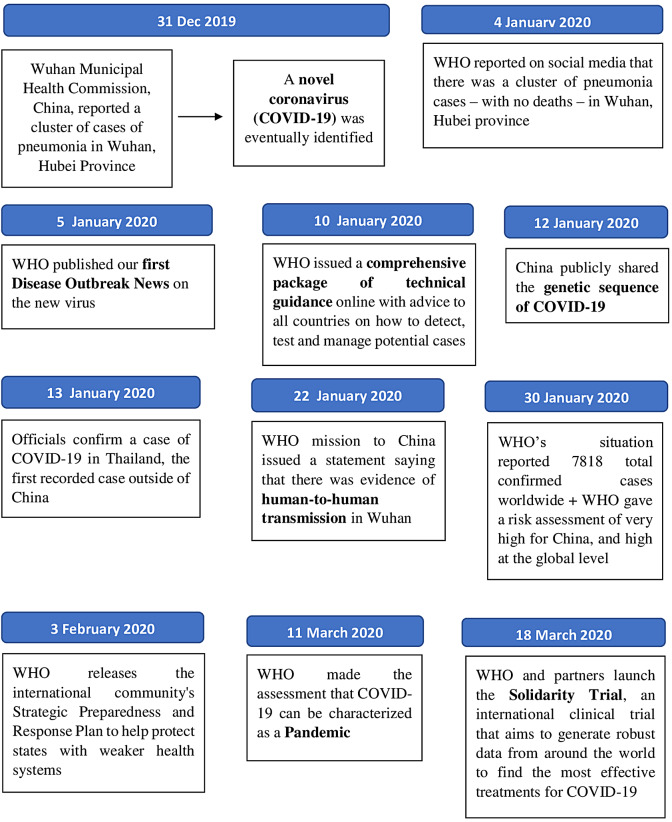
WHO timeline frame for COVID-19.

By the end of 2019, the novel coronavirus (COVID-19) has become a public health threat to people all over the world,^2^ with transmission occurs primarily through respiratory droplets and indirectly via contaminated surfaces.^3^ On March 11, 2020, the World Health Organization (WHO) declared the ourbreak of COVID-19 as a global pandemic.^4^ A WHO statement^5^ of the timeline frame for COVID-19 is shown in Figure 1.

The exact incidence of neurological complications from COVID-19 infection remains unknown. Neurological symptoms were sought to be more common with the severe form of the disease,^6^ however, recently, neurologic symptoms are common in early stages of the disease and are the second most common symptom after respiratory symptoms.^3^ The most common neurological manifestations in COVID-19 patients include headache, confusion, dizziness, mild cognitive impairment, altered taste, loss of smell, blurred vision, as well as muscle and nerve pain.^7^ Several neurological and neuroradiological phenotypes have been observed in patients with COVID-19 despite the short duration of the current pandemic.

Limited pathological autopsy results are available for COVID-19 patients and showed not only lung affection, but also the involvement of other organs including the heart, liver, kidneys, spleen, hilar lymph nodes, bone marrow, and even brain tissues.^8^ Through neuro-invasion, the virus can affect both neurons and glial cells and induce a wide range of neurological pathologies. Various pathogenic mechanisms have been proposed for the effects of COVID-19 on the nervous system including: (1) direct central nervous system (CNS) invasion through the blood or lymphatic system, (2) regulation of angiotensin II converting enzyme (ACE2) receptor, and (3) hypoxia and immune-mediated neurological damage.^6^ Both clinical syndromes SARS-CoV and MERS-CoV can directly cause brain damage in experimental animals and real patients.^9^ A summary of possible neurological syndrome/manifestations pathogenesis in COVID-19 patients is demonstrated in Table 1.

**Table 1.** Possible Pathogenesis of Neurological Manifestations Among COVID-19 Patients.

**Neurological Manifestation, Disorder, or Syndrome**	**Possible Pathogenic Mechanism(s)**
Dizziness	Nonspecific and possibly systemic neurological symptoms
Headache	Nonspecific and possibly systemic neurological symptoms Role of ACE2 in the pathophysiology has recently introduced
Confusion DCL Encephalopathy	Severe hypoxia in severe COVID-19 patients Brain edema ➔ detected in autopsies of patients who died from COVID-19 Effect of drugs
Taste impairment Smell impairment	Direct invasion of nasal mucosa Post viral olfactory/taste dysfunction Specific tropism of these viruses for structures of the olfactory/taste sensory epithelium
Cerebrovascular disease/stroke	Marked inflammatory and procoagulant response (increased of CRP and D-dimer) SARS-CoV-2 binds to ACE2 receptors on endothelial cells ➔ increase in blood pressure Cytokine storm syndrome may be another risk factor
Peripheral nerve disorders (eg, GBS)	Increased CSF proteins without cells Possibly immune-mediated Molecular mimicry between specific viral proteins and proteins on peripheral nerves (gangliosides)
Ataxia	Possibly immune-mediated
Seizure	Metabolic derangements Effect of drugs
Rhabdomyolysis Myalgia	Cytokine storm syndrome
Acute hemorrhagic necrotizing encephalopathy	Cytokine storm syndrome

Abbreviations: ACE, angiotensin converting enzyme; CRP, C-reactive protein; CSF, cerebrospinal fluid; DCL, disturbed conscious level; GBS, Guillain-Barre syndrome.

The most common respiratory viruses pathogenic to humans are influenza, respiratory syncytial virus, human metapneumovirus, and coronavirus. These virsuses adversely affect numerous systems in the human body. Severe respiratory diseases caused by these viruses are associated with various neurological symptoms. For example, respiratory syncytial virus has been detected in the cerebrospinal fluid (CSF) of patients and known to cause encephalitis, epileptic seizures, and ataxia. In previous studies, cases of influenza virus were reported to have various neurological complications including CNS manifestations such as encephalitis, meningitis, and myelitis as well as PNS manifestations such as Guillain-Barré syndrome (GBS).^6^ ^,^^10^

Human-to-human transmission occurs through coughing or sneezing with spread of respiratory droplets. It can also be transmitted by close contact between humans,^1^ since the virus is highly contagious and has a relatively long latency period.^11^ COVID-19 usually runs an insidious clinical course in two phases; an initial incubation phase and later a clinical symptomatic phase. During the initial incubation phase (3-5 days), the virus is attempting to seed at the most peripheral and inferior parts of the lungs.^7^ In the clinical phase, the virus primarily causes severe respiratory symptoms as fever, cough, and fatigue. Other symptoms include headache, hemoptysis, and dyspnea.^12^ These symptoms can culminate in a cytokine storm which later leads to acute respiratory distress syndrome (ARDS).^13^

From a diagnostic point of view, the WHO recommends collecting samples from both the upper and lower respiratory tracts to be assessed for viral RNA using polymerase chain reaction (PCR). However, a strong clinical suspicion with negative test warrants re-testing.^1^ Diagnosis of COVID-19 remains challenging for two reasons; the first reason is the absence of clear and specific clinical symptoms for the disease, and the second one is that some patients are unaware of being infected as they do not suffer from fever or respiratory symptoms although they are contagious during the incubation period.^14^

A consensus has been issued for neurologists to be involved in the prevention and management of COVID-19; this indicates that COVID-19 patients may present with neurological symptoms first. Hence, neurologists and other healthcare providers should pay close attention to these manifestations and have a high index of suspicion when evaluating patients in an endemic area. Early recognition may help in the initiation of proper management strategies such as early isolation to prevent spreading of the virus and avoid clinical deterioration of patients.^11^

The aim of this review is to focus and summarize the available published data from inception on neurological manifestations in patients with COVID-19.

## Methods

We followed the Preferred Reporting Items for Systematic Reviews and Meta-Analyses (PRISMA) statement guidelines^15^ during the preparation of this review.

### Eligibility criteria

#### Inclusion criteria

**Trial or study type:** Articles publishing findings from randomized controlled trials (RCT), non–randomized controlled trials, single arm studies, observational studies, cross sectional studies, retrospective studies, case reports, and case series of neurological manifestations in patients with COVID-19 were included.

**Population:** Patients with COVID-19 clinically and/or laboratory-diagnosed and those presented with neurological manifestations (with or without other manifestations) of any age group and any gender were included. Patients with primary neurological disorders developed or complicated by COVID-19 were excluded.

Neurological manifestations were divided into: (1) CNS manifestations including dizziness, headache, confusion, encephalopathy, disturbed conscious level (DCL), acute cerebrovascular disease, myelopathy/myelitis, pure meningitis, meningo-radiculitis, ataxia, and seizure and (2) PNS manifestations including taste impairment, smell impairment, vision impairment, isolated cranial nerve impairment, nerve pain/neuropathic pain, polyradiculitis, GBS, Miller fisher syndrome, peripheral neuropathy, muscles disease, and myasthenic syndrome. Encephalopathy included all types of parenchymal lesions such as vasculitis, stroke, multiple sclerosis, CNS sarcoidosis, and posterior reversible encephalopathy syndrome. Neuropathy included all types of peripheral involvement (mononeuropathy, mononeuritis multiplex, and polyneuropathy). Muscle diseases included myopathy (except congenital or primary myopathy) and all types of myositis (polymyositis, dermatomyositis, and inclusion body myositis). Patients with muscle pain/myalgia without muscle weakness and/or neurophysiological diagnosis of myopathy/myositis were excluded.

**Exclusion criteria:** Non-English language studies, animal studies, studies with full text articles unavailable, theses, conference papers, and ongoing trials were excluded. Articles that were published before the era of SARS-Cov-2 (i.e., before December 2019) were excluded.

***Objective:*** To systematically assess the neurological manifestations in patients diagnosed with COVID-19.

***Study design*:** This is a systematic review.

### Sources of information and search

Medical electronic databases: PubMed, Scopus, and Cochrane; from inception to May 2020 were searched for all published studies using a combination of keywords, MESH terms, and text words, including: “COVID 19” and “neurological disorders.” An independent search of Google Scholar was also performed to ensure that no additional clinical trials were missed. To ensure literature saturation, the author scanned the reference lists of the included studies or relevant reviews identified through the search. In case of duplicate publications, only the most recent and updated report that includes the clinical trial’s full data were included.

A separate search was done to learn more about the ongoing trials on neurological manifestations in patients with COVID-19. We searched clinicaltrials.gov “www.clinicaltrials.gov” (using the same previous search queries) for the following types of ongoing clinical trials: not yet recruiting, recruiting, enrolling by invitation, as well as active and not recruiting trials.

### Selection of studies

The author selected eligible studies. Screening of search results was performed in two steps:

- Screening titles and abstracts against the selection criteria. Articles which were not clear from their titles or abstracts were reviewed against the selection criteria through their full text.
- Retrieving and screening the full-text articles of eligible abstracts for eligibility to systematic review.

### Data extraction

The author extracted data independently using an online data extraction form. These data included study authors, year, study type, sample size, mean age, method of COVID-19 diagnosis, neurological symptoms, severity of disease, investigations, and management.

Data were exported from the online form as a MS excel sheet.

### Statistical analysis

The mean age and standard deviation (SD) were calculated for all patients using pooled mean and pooled SD equation according to the Cochrane handbook of systematic reviews of interventions 5.1.0 (updated March 2011).^16^ When data were expressed as median and interquartile range, we used the equation of Hozo et al^17^ at BMC Research Methodology to calculate or estimate mean and SD.

Data were expressed as means with SD (unless stated otherwise). Statistical results were considered to be significant when the *P*-value was less than .05.

Data analysis was performed using the SPSS Statistical Package v23 (IBM Corp., Armonk, NY).

### Synthesis of data and analysis

Due to heterogeneity and low sample size of studies, no statistically justified analyses could be performed on the provided data. Hence, a descriptive analysis of the published studies was performed instead.

### Summary measures

The search strings, list of relevant reviews, data coding, and quality criteria used in this review can be requested from the corresponding author.

### Patient and public involvement

Patients were not involved in setting the research question or outcome measures, developing plans for design, or implementing the study. Also, no patients were asked to advise on the interpretation or writing up of results. There are no plans to disseminate the results of this research to study participants or the relevant patient community.

## Results

### Selection of articles

**Figure 2.**
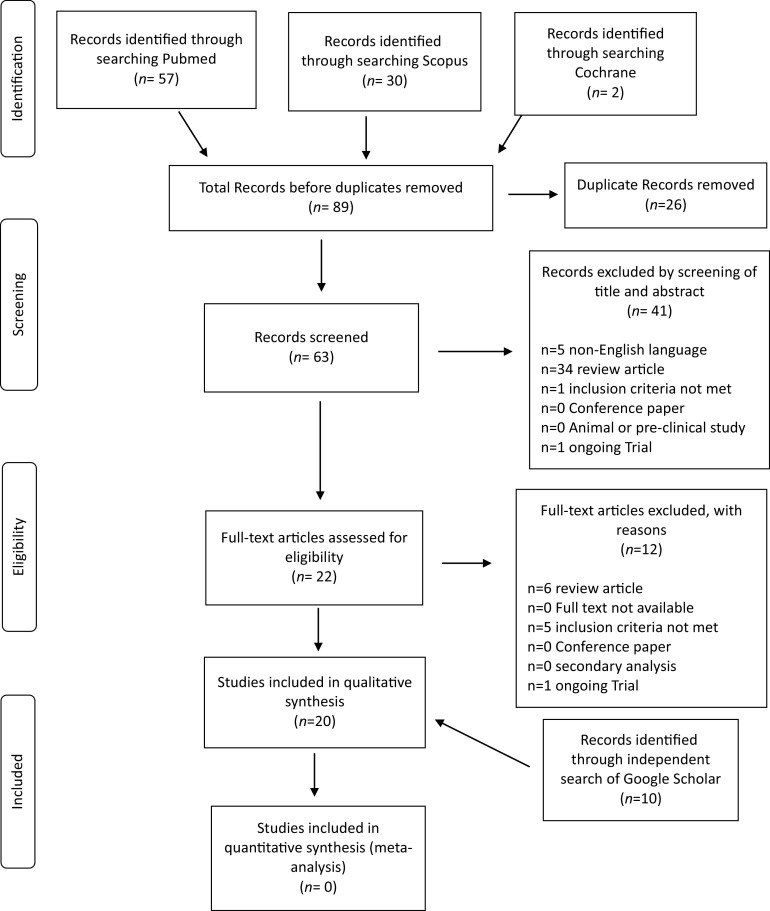
PRISMA Flowdiagram of included and excluded articles in the systematic literature search.

The systematic literature search and subsequent selection are summarized in a flow diagram (Figure 2). The PubMed database online search identified 57 publications, a second database search through Scopus identified 30 publications, and a third search through Cochrane database identified 2 publications. After 26 duplicate publications were removed, a total of 63 publications were screened for title and abstract. All articles with animal instead of human patients, review articles and non-English language articles were excluded (n = 41 articles). A total of 22 full-text articles were assessed for eligibility, with 12 publications excluded for the following reasons: review articles (n = 6), ongoing trials (n = 1), and non–compliance with the inclusion criteria (n = 5).

An independent search of Google Scholar was also performed and identified 11 publications. Three publications were case reports and the remaining eight publications were retrospective observational studies.

To learn more about the ongoing trials on neurological manifestations in patients with COVID-19, we searched clinicaltrials.gov. A total of 31 trials were assessed for eligibility, with 17 publications excluded for noncompliance with the inclusion criteria.

### Study characteristics

A summary of the included studies is demonstrated in Table 2a. A total of 20 studies from inception to May 2020 were included in the descriptive analysis and systematic review. Case reports and case series represented the majority of studies (n = 11), while the remaining nine studies were retrospective observational studies. In this review, we focused on the nine retrospective observational studies excluding case reports and case series due to lack of sufficient data and to avoid bias.

**Table 2a.** Summary of Included Studies.

**Study ID**	**Study Type**	**Number of Patients (Neurology/Total)**	**COVID-19 Diagnosis**	**Neurological Manifestation/Disorder**
Shao et al (2020)	Retrospective observational study	n = 9/136	Clinical and laboratory diagnosis*	-Headache/dizziness
Mao et al^18^	Retrospective observational study	n = 72/214	Clinical and laboratory diagnosis	-CNS manifestations: dizziness, headache, DCL, acute cerebrovascular disease, ataxia, and seizure -PNS manifestations: taste impairment, smell impairment, vision impairment, and nerve pain
Guan et al (2019)	Retrospective observational study	n = 150/1099	Clinical and laboratory diagnosis	-Headache
Wang et al (2020)	Retrospective observational study	n = 22/138	Clinical and laboratory diagnosis	-Headache (n = 9) -Dizziness (n = 13)
Yang et al (2020)	Retrospective observational study	n = 3/52	Clinical and laboratory diagnosis	-Headache
Huang et al^19^	Retrospective observational study	n = 3/38	Clinical and laboratory diagnosis	-Headache
Chen et al^20^	Retrospective observational study	n = 17/99	Clinical and laboratory diagnosis	-Headache (n = 8) -Confusion (n = 9)
Li et al^9^	Retrospective observational study	n = 13/221	Clinical and laboratory diagnosis	-Stroke: acute ischemic stroke (n = 11), CVST (n = 1), and cerebral hemorrhage (n = 1)
Sun et al^21^	Retrospective case series study (in pediatric patients)	n = 1/8	Clinical and laboratory diagnosis	-Headache

Abbreviations: CNS, central nervous system; DCL, disturbed conscious level; PNS, pheripheral nervous system.

A summary of the included ongoing trials (n = 14) are demonstrated in Table 3. Seven studies started recruiting (50%), 1 study was enrolling subjects by invitation, and the remaining 6 studies were not yet recruiting subjects. Only 5 studies were multi-center studies (35.7%) and the remaining studies were single-center studies (n = 9).

### Baseline characteristics of included studies

The baseline characteristics of neurological manifestations are demonstrated in Table 4. A total of 2005 patients participated in the nine studies included in this systematic review. A total of 290 COVID-19 patients with 14.5% incidence of neurological manifestations were included in our review after omitting case reports to get unbiased data of the overall incidence.

A total of 10 neurological symptoms were reported in the 9 studies included in this review. Symptoms were subdivided into CNS symptoms (n = 6) and PNS symptoms (n = 4). CNS symptoms were more common, representing 91% of all neurological patients, while PNS symptoms represented only 9%. Also, the incidence of CNS manifestations was higher than that of PNS manifestations, with 9.1% in the former and 3.6% in the latter.

Six neurological symptoms including ataxia, seizure, nerve pain, smell, taste, and visual impairment were reported in one study only, dizziness was reported in three studies, and stroke and confusion/DCL were reported in two studies.

Headache represented the most common neurological symptom in terms of number of patients (n = 211) and number of studies (n = 8), meanwhile dizziness has the highest incidence (11.9%) amongst COVID-19 patients.

Neurological manifestations were divided according to COVID-19 severity into: (1) nonsevere manifestations which include: mild, moderate symptoms or non-ICU/ward admission and (2) severe manifestations which include severe symptoms or ICU admission. All CNS manifestations were more frequent in severe COVID-19 patients except headache which was more frequent in nonsevere COVID-19 patients (72.4%). On the other hand, PNS manifestations showed variable results where visual impairment and nerve pains were more frequent in the severe COVID-19 category of patients with percentages of 66.7% and 80%, respectively, while smell and taste impairment, which are highly common PNS manifestations, were more frequent in the nonsevere category with percentages of 27.3% and 25%, respectively. Only one study did not categorize neurological manifestations according to COVID-19 disease severity.

All the included studies were carried out on adult patients except one study which was performed in pediatric patients with limited number of participants (n = 8).

No data regarding the mortality of COVID-19 patients with neurological manifestations could be extracted from the included studies in our systematic review.

### Baseline characteristics in case reports

Although the data from case reports were not included in our statistical results to avoid getting biased incidence and results, it was important to analyze them for better definition of the spectrum of neurological manifestations in COVID-19 patients (Table 2b).

**Table 2b.** Summary of Included Case Report and Case Series Studies.

**Study ID**	**Study type**	**COVID-19 Diagnosis**	**Neurological Manifestation/Disorder**	**Investigations**	**Management**
Liu et al^22^	Case report (n = 1)	Clinical diagnosis only, negative laboratory test	-Vertigo	-Chest CT: GGO in left lung -18F-FDG PET/CT: multiple GGO in both lungs, with increased tracer uptake in the whole colon -Virus antigen test: negative	-Antibiotics -Antiviral -Anti-inflammatory -Passive immunization
Yin et al^8^	Case report (n = 1)	Clinical and laboratory diagnosis (+ve throat swab)	-DCL + positive signs of meningeal irritations + signs of pyramidal affection	-Chest CT: multiple GGO with multiple fibrous cord-like shadows in both lungs -Throat swab: positive for COVID-19 -CT brain: NAD -CSF analysis: ++ protein, −− glucose, cell count = 1 × 10^6^/L	-Ribavirin antiviral therapy -Arbidol -Traditional Chinese medicine
Dinkin et al^23^	Case report (n = 2)	Clinical and laboratory diagnosis (+ve nasal swab)	-Miller Fisher syndrome (n = 1) -Ophthalmoparesis from cranial nerve palsy (n = 1)	-Nasal swab: positive for SARS-CoV-2 -CXR: normal in case 1, bilateral airspace opacities in case 2	-IV Ig 2 g/kg over 3 d (case 1) -hydroxychloroquine for COVID-19, no specific treatment for neurological manifestations with no recovery (case 2)
Sohal et al^24^	Case report (n = 1)	Clinical and laboratory diagnosis	-Seizure	-PCR for SARS COV-2 was positive -Chest CT: bibasilar opacities -Brain CT: microvascular ischemic changes	-Oseltamivir -Hydroxychloroquine and azithromycin + vancomycin and piperacillin tazobactam -Loading dose of levetiracetam followed by maintenance dose, then added Valproate (due to recurrence of seizures)
C’amara Isabel et al. (2020)	Case report (n = 1)	Clinical and laboratory diagnosis (+ve nasal swab)	-Seizure in neonate	-Nasal swab: positive for SARS-CoV-2 -EEG: continuous background pattern with sleep–wake cycles	-Empirical antibiotherapy
Gutiérrez-Ortiz et al^25^	Case report (n = 2)	Clinical and laboratory diagnosis (+ve oropharyngeal swab and PCR)	-Miller Fisher Syndrome (n = 1) -polyneuritis cranialis (n = 1)	-Oropharyngeal swab: positive for SARS-CoV-2 -PCR: positive for SARS-CoV-2 -CSF: negative for SARS-CoV-2	-IV Ig 0.4 g/kg for 5 d (case 1) -Recovery on nonspecific medications (case 2)
Filatov et al^26^	Case report (n = 1)	Clinical and laboratory diagnosis^a^	-Encephalopathy	-CT brain: old area of hypodensity in the right temporal region -EEG: bilateral slowing and focal slowing in the left temporal region with sharply countered waves -Chest CT: patchy bibasilar consolidations and subpleural opacities	-Vancomycin, meropenem, and acyclovir. -AED -Hydroxychloroquine and lopinavir/ritonavir
Zanin et al^27^	Case report (n = 1)	Clinical and laboratory diagnosis (PCR)	-Demyelinating CNS disorder ➔ DCL, seizures	-PCR: positive for SARS-CoV-2 -Chest CT: interstitial pneumonia -CT brain: normal -MRI brain: alterations of the periventricular white matter, hyperintense in T2WI -CSF: normal, negative for SARS-CoV-2	-AED: lacosamide, levetiracetam, and phenytoin -Dexamethasone 20 mg/d for 10 d, then 10 mg/d for 10 d
Poyiadji et al^28^	Case report (n = 1)	Clinical and laboratory diagnosis (+ve Nasopharyngeal swab)	-Acute necrotizing hemorrhagic encephalopathy	-Nasopharyngeal swab: positive for SARS-CoV-2 -CSF: no bacterial growth, negative virology analysis -CT Brain: symmetric hypoattenuation within the bilateral medial thalami -CT angiogram and CT venogram: normal	-IV Ig^b^
Ye et al^29^	Case report (n = 1)	Clinical and laboratory diagnosis^a^	-Encephalitis	-Chest CT: multiple subpleural GGOs -CT Brain: normal -CSF: normal, negative for SARS-CoV-2	-Supportive treatment only including mannitol infusion
Avula et al^30^	Retrospective Case series (n = 4)	Clinical and laboratory diagnosis	-Stroke	-CT Brain: acute infarct of the left MCA territory (case 1), acute right frontal lobe infarction (case 2), acute right MCA infarction (case 3) -Chest CT: diffuse GGOs (case 1) -CXR: bilateral peripheral opacities (case 2) -PCR: positive for SARS-CoV-2 -MRI brain: acute infarct in left medial temporal lobe (case 4)	-Conservative medical treatment for acute stroke
Beyrouti et al^31^	Retrospective Case series (n = 6)	Clinical and laboratory diagnosis	-Stroke	-CT chest: Bilateral GGO change and consolidation -MRI brain: acute left vertebral artery thrombus and acute left posterior-inferior cerebellar artery territory infarction (patient 1), acute infarction in the right corpus striatum (patient 4), acute infarction in the right thalamus, left pons, right occipital lobe and right cerebellar hemisphere (patient 6) -CT brain: acute right parietal cortical and left cerebellar infarct with mass effect and hydrocephalus (patient 2), thrombus in the left posterior cerebral artery and acute infarction in the left temporal stem and cerebral peduncle (patient 3), thrombotic occlusion of a proximal M2 branch of the right middle cerebral artery (patient 5)	-Therapeutic LMWH anticoagulation -IV thrombolysis (patients 5 and 6)

Abbreviations: ++, increase; −−, decrease; AED, anti-epileptic drug; CNS, central nervous system; CSF, cerebrospinal fluid; CT, compterized tomograpgy; CVST, cerebral venous sinus thrombosis; DCL, disturbed conscious level; EEG, electroencephalography; GGO, ground-glass opacities; Ig, immunoglobulins; IHCA, in-hospital cardiac arrest; IV, intravenous; LMWH, low molecular weight heparin; MCA, middle cerebral artery; MRI, magnetic resonance imaging; PCR, polymerase chain reaction; PET, positron emission tomography; PNS, peripheral nervous system; SAH, subarachnoid hemorrhage.

a Exact method of laboratory diagnosis was not mentioned.

b Exact dose not mentioned.

**Table 3.** Summary of Ongoing Trials of Neurological Manifestations in COVID-19 Patients.

**Study ID**	**Study Title**	**Study Type**	**Study Status**	**Country**	**Study Objective/Aim**
1	Testing for dysautonomia in patients hospitalized for SARS-CoV-2 infection (COVID-19): COVIDANS study	Prospective cohort study	Recruiting	France, single center	To record the long-term variability in heart rate, reflecting autonomic balance, of patients screened positive for SARS-CoV-2
2	COVID-19 prevalence and cognitive deficits in neurological patients	Prospective cohort study	Not yet recruiting	Denmark, multicenter	To investigate the COVID-19 prevalence, associated morbidity and long-term cognitive deficits in consecutive patients presenting with acute neurological symptoms
3	Cohort of patients with COVID-19 presenting neurological or psychiatric disorders (CoCo-neurosciences)	Retrospective cohort study	Not yet recruiting	France, single center	Impact of neurological or psychiatric manifestations in patients with COVID-19 infection
4	Neurologic manifestations of COVID-19 in children	Prospective cohort study	Recruiting	United States, multicenter	Frequency of neurologic manifestations of COVID 19 among pediatric patients requiring hospital admission for confirmed or presumed COVID 19
5	Frequency and clinical evolution of olfactory and taste disorders in COVID-19 patients	Retrospective study	Enrolling by invitation	France, single center	To evaluate prevalence of anosmia and dysgeusia in COVID-19 diagnosed patients
6	Neurodegeneration markers and neurological course in severe Covid-19 infection	Prospective, single group study	Not yet recruiting	France, single center	Examine if indirect signs of CNS lesion are observed in association with severe Covid-19 infection using neurodegeneration markers level
7	Qualitative and quantitative evaluation of anosmia over time in clinically symptomatic patients tested for COVID-19 infection	Prospective cohort study	Recruiting	France, single center	To describe the prevalence of olfactory and gustatory dysfunction and assess the factors associated with positive SARS-CoV-2 infection
8	Acute encephalopathy in critically ill patients with COVID-19	Prospective cohort study	Recruiting	United States, France, multicenter	Reporting the prevalence of acute encephalopathy at initial management in Intensive care or neurocritical care
9	Finding out if COVID-19 Infection can be predicted by changes in smell and/or taste	Prospective cohort study	Recruiting	United Kingdom, single center	To compare case fatality rate in COVID-19 positive with documented history of loss/reduced sense of smell and/or taste compared to COVID-19 positive with documented history of no loss/reduced sense of smell and/or taste
10	Trial evaluating the efficacy of local budesonide therapy in the management of hyposmia in COVID-19 patients without signs of severity	RCT	Not yet recruiting	France, single center	Given budesonide nasal spray to treat hyposmia associated with COVID-19
11	Anosmia rehabilitation in patients post coronavirus disease (COVID 19)	RCT, 2 arm +1 control	Not yet recruiting	Canada, single center	Budesonide with the nasal irrigations as treatment
12	Neurocognitive impairment in patients with COVID-19	Case–control prospective study Follow-up after 3 mo	Recruiting	Germany, multicenter	Using biomarkers of neuroaxonal injury and scales to assess the neurocognitive performance of study participants
13	Prospective Registry of Corona Virus Disease 2019 (COVID-19) patients with neuromuscular involvement	Prospective cohort study	Recruiting	Germany, Single center	Multimodal assessment of neuromuscular pathology associated with SARS-CoV-2 infection
14	Chronic fatigue etiology and recovery in COVID-19 patients: the role of fatigability	Nonrandomized trial	Not yet recruiting	France, multicenter	To determine whether deteriorated neuromuscular function is involved in the feeling of fatigue of COVID-19 patients

Abbreviations: ARDS, acute respiratory distress syndrome; COVID-19, novel coronavirus; CRS, cytokine release syndrome; RCT, randomized controlled trial; SARS-CoV-2, severe acute respiratory syndrome coronavirus 2.

**Table 4.** Baseline Characteristics of Neurological Manifestations in the Included Studies.^a^

**Neurological Manifestations/Disorder**	**Study Type (no.)**	**Number of Patients Neurological/Total (%)**	**COVID-19 Severity Category No. (%)**
Dizziness	Retrospective observational study (n = 3)	58/488 (11.9%)	Nonsevere: 24 (41.4%) Severe: 34 (58.6%)
Headache	Retrospective observational study (n = 8)	211/1784 (11.8%)	Nonsevere: 147 (72.4%)^a^ Severe: 56 (27.6%)
Confusion/DCL	Retrospective observational study (n = 2)	25/313 (8%)	Nonsevere: 3 (18.8%)^a^ Severe: 13 (81.2%)
Taste impairment	Retrospective observational study (n = 1)	12/214 (5.6%)	Nonsevere: 9 (75%) Severe: 3 (25%)
Smell impairment	Retrospective observational study (n = 1)	11/214 (5.1%)	Nonsevere: 8 (72.7%) Severe: 3 (27.3%)
Cerebrovascular disease/stroke	Retrospective observational study (n = 2)	19/435 (4.4%)	Nonsevere: 1 (5.3%) Severe: 18 (94.7%)
Nerve pains	Retrospective observational study (n = 1)	5/214 (2.3%)	Nonsevere: 1 (20%) Severe: 4 (80%)
Vision impairment	Retrospective observational study (n = 1)	3/214 (1.4%)	Nonsevere: 1 (33.3%) Severe: 2 (66.7%)
Ataxia	Retrospective observational study (n = 1)	1/214 (0.5%)	Nonsevere: 0 Severe: 1 (100%)
Seizure	Retrospective observational study (n = 1)	1/214 (0.5%)	Nonsevere: 0 Severe: 1 (100%)

NB: COVID-19 severity either (1) nonsevere includes: mild, moderate symptoms, or non-ICU/ward patients or (2) severe includes: severe symptoms or ICU patients.

Abbreviations: DCL, disturbed conscious level; no., number.

a Chen et al (2020) study not reported severity of COVID-19 according to neurological symptoms, so patients were excluded from analysis in severity column.

Case reports and case series (n = 12 studies) reported a total of 22 patients with neurological manifestations. CNS manifestations represented the majority of reported neurological symptoms in case reports (n = 18) (82%), while PNS manifestations were reported in 4 cases only^23^ ^,^^25^ (18%).

PNS manifestations required treatment with intravenous (IV) immunoglobulins (Ig)^25^ ^,^^28^ in addition to the COVID-19 treatment. Other manifestations were treated with nonspecific treatments including antibiotics, antiviral, and other symptomatic treatments as antiepileptic drugs (AED) for seizures^24^ or antiplatelets, IV thrombolysis and low molecular weight heparin (LMWH) for stroke patients,^31^ while steroids (dexamethasone) were used once for a case of demyelinating CNS disorder.^27^

In all of the included case reports, COVID-19 diagnosis was done both clinically and by PCR, except in only one case report by Liu et al,^22^ where the diagnosis was based on the clinical examination supported by laboratory tests and imaging with negative antigen. In other two reports,^26^ ^,^^29^ the exact laboratory diagnostic method was not mentioned.

One case report reported neurological manifestations in a neonate^32^ with COVID-19, while the remaining studies (n = 11) reported them in adult patients.

## Discussion

This systematic review differs from previous reviews^6^ ^,^^12^ in many points. First, it comprised all the published literature on neurological manifestations in COVID-19 patients including analysis and summarization of case reports in addition to the most recent published data (till May 2020). Second, ongoing trials on the neurological manifestations in COVID-19 patients were searched, summarized, and analyzed. Third, neurological manifestations were summarized in detail in terms of symptomatology apart from each study analysis. The strengths and limitations to this review in detail as well as the implications for future studies in this field are discussed later in the paper.

Neurological manifestations can be nonspecific or mild at the early stage of COVID-19 infection.^11^ A growing number of studies reported this type of manifestations in COVID-19 patients which raises the researcher’s curiosity to study them in depth. The aim of this review is to focus on and summarize the available published data as well as ongoing trials on neurological manifestations in patients with COVID-19.

The incidence of neurological manifestations in our review was 14.5%. A review by Carod-artal^6^ concluded that the exact incidence of neurological manifestations in SARS-CoV-2 patients is unknown. Another review by Asadi-pooya et al^12^ marginalized its results regarding the percentage of CNS manifestations incidence (25%) in hospitalized patients in a Chinese study.^18^ A review by Jin et al^11^ stated that more than one-third of COVID-19 patients experienced various degrees of neurological symptoms.

Regarding the neurological manifestations subgroup in our review, dizziness and headache were the most common CNS manifestations among patients with percentages of 11.9% and 11.8%, respectively, followed by confusion/DCL (8%). In PNS manifestations, taste and smell impairments were more common among patients with percentages of 5.6% and 5.1%, respectively. These findings are in line with Jin et al^11^ review which described dizziness and headache in CNS manifestations as well as and taste and smell impairments in PNS manifestations as the most common symptomatology.

All the included studies categorized neurological manifestations according to COVID-19 disease severity except one study by Chen et al.^20^ In our review, CNS manifestations were more frequent in severe COVID-19 patients except headache. In PNS manifestations, visual impairment and nerve pain were more frequent in the severe category of patients with percentages of 66.7% and 80%, respectively, while smell and taste impairments, which are highly common PNS manifestations, were more frequent in the nonsevere category with percentages of 27.3% and 25%, respectively. Previous reviews^6^,^11^ mentioned that neurological symptoms are more likely to occur in patients with the severe form of COVID-19 compared to those with milder disease. It was also reported previously that the most common neurological manifestations in severe COV-2 infection are stroke and DCL.

Pediatric age group was discussed only in one study by Sun et al^21^ with a limited number of participants (n = 8). Most of the studies in literature investigated neurological manifestations in adult COVID-19 patients. A case report^32^ in our review discussed a case of male neonate (26 days old) with COVID-19 presenting with febrile seizures. This is due to rarity of reports on COVID-19 in the pediatric age group since it seems to be uncommon in children,^2^ ^,^^19^ ^,^^33^ which may be attributed to the lower outdoor activity of children compared to adults including international travels.^2^

As previously mentioned, various respiratory viruses are associated with neurological manifestations. Human coronavirus (HCoV) is an example which reaches the CNS through olfactory bulb causing inflammation and demyelination. After the infection is set in the respiratory tract, the virus reaches the CSF and brain in less than 7 days, causing various neurological complications including CNS manifestations as encephalitis, meningitis, myelitis as well as PNS manifestations as GBS. This explains the neurological manifestations encountered in our case reports including (a) CNS affection as: (1) demyelinating CNS disorder in a study by Zanin et al,^27^ which was successfully treated by steroids (dexamethasone), (2) acute necrotizing hemorrhagic encephalopathy in a study by Poyiadji et al,^28^ which was treated by IV Ig with no reported response to medication, (3) encephalitis in a study by Ye et al^29^ with no specific treatment, (4) stroke in a study by Avula et al^30^ with no intervention or conservative medical treatment for acute stroke, and (5) seizures in a study by Sohal et al,^24^ which were treated by antiviral and AED but unfortunately the patient died and (b) PNS affection as: (1) Miller Fisher syndrome in two studies^23^ ^,^^25^ which was successfully treated by IV Ig and partially improved (2) polyneuritis cranialis in a study by Gutiérrez-Ortiz et al,^25^ which was resolved completely on nonspecific treatment with acetaminophen, (3) ophthalmoparesis in a study by Dinkin et al,^23^ which was treated by hydroxychloroquine for COVID-19 and the neurological symptoms were gradually resolved.

Only in one case report by Liu et al,^22^ COVID-19 diagnosis was based on clinical symptoms supported with computerized tomography (CT) chest inspite of the negative antigen testing. Although PCR for SARS-COV-2 is the gold standard method and corner stone in the diagnosis of COVID-19, diagnosis can be made if there is a high clinical suspicious as the infected people may or may not have abnormal laboratory findings or lung changes on CT chest and negative nucleic acid test,^11^ which renders re-testing^1^ in such cases. This is explained by the low test sensitivity of around 70%.^34^ In a case series by Fang et al,^35^ it was found that the sensitivity of chest CT was higher than that of PCR (98% vs 71%, respectively).

Mortality in patients with neurological disorders were not observed in the studies included in our analysis. The mortality rates differ across different pathogenic types SARS-CoV, MERS-CoV, and SARS-CoV-2 with rates of 9.6%, 34.4%, and 5.3% (till April 3, 2020), respectively.^36^ The difference in mortality rate between COVID-19 patients with and without neurological manifestations needs further studies since there are no available published data. However, a previous review^6^ stated that neurological symptoms are more common with the severe form of COVID-19.

### Strengths and limitations of the review

The first strength of this review is that it identifies the gaps in our current knowledge about neurological manifestations in COVID-19 patients. Second, the review comprises all the available articles from inception (December 2019) till May 2020 regarding this issue. Third, it can serve as a model for future studies investigating the neurological manifestations in COVID-19 patients and their proper management. Fourth, it included search and summarizization of ongoing trials about neurological manifestations inpatients with this disease.

Research in this topic has important limitations that should be discussed. First, studies significantly vary in samples, methodologies, and measured outcomes. Second, neurological manifestations were not studied in depth in the available studies and were reported with other symptoms with no specific scales or scores to assess such manifestations or disorders. Third, the incidence of neurological manifestations was extracted only from observational studies since it cannot be deducted from case reports which represent the majority of studies, in terms of number, reporting neurological manifestations. Fourth, all studies did not report a specific management of neurological manifestations in COVID-19 patients except for case reports; however, some of them also did not report a specific treatment. Fifth, all studies were conducted on adult patients except one study with a limited number of patients. Finally, another limitation is the lack of duration of neurological manifestations, since, It is now known that some patients continue to have symptoms for a relatively long time after resolution of their COVID-19 symptoms.

Furthermore, a difficulty which was encountered in the current systematic review is that most of the studies reviewed were case reports, case series, and observational studies with limited number of COVID-19 patients with neurological manifestations and that most of studies included were from china.

### Implications for future research

In future studies, more suitable RCTs need to be conducted to determine the spectrum of neurological manifestations in COVID-19 patients and their prognosis and mortality rates. Also, further studies on pediatric COVID-19 patients are needed to define the spectrum of neurological manifestations among this age group.

Long follow-up periods are needed to detect neurological complications in post COVID-19 patients.

Additional studies are needed to get accurate information about the incidence of neurological manifestations in COVID-19 patients with the development of international consensus/guidelines for proper management of such manifestations.

## Conclusions

The purpose of this review was to summarize the available published data and ongoing trials on the neurological manifestations in patients with COVID-19.

From the descriptive analyses and available data of relatively small sample-sized studies, it can be concluded that in spite of the aforementioned limitations, a wide spectrum of neurological manifestations including CNS and PNS manifestations can occur in COVID-19 patients whatever the severity of the disease is, with higher incidence among the severe category of patients. A detailed history and neurological examination with proper assessment by expert neurologists is needed in the evaluation of patients with COVID-19 and neurological symptoms.
